# Transcriptome analysis of neural progenitor cells derived from Lowe syndrome induced pluripotent stem cells: identification of candidate genes for the neurodevelopmental and eye manifestations

**DOI:** 10.1186/s11689-020-09317-2

**Published:** 2020-05-11

**Authors:** Hequn Liu, Jesse Barnes, Erika Pedrosa, Nathaniel S. Herman, Franklin Salas, Ping Wang, Deyou Zheng, Herbert M. Lachman

**Affiliations:** 1 Department of Genetics, Albert Einstein College of Medicine, Bronx, New York, USA; 2 Department of Psychiatry and Behavioral Sciences, Albert Einstein College of Medicine, Bronx, New York, USA; 3 Dominick P Purpura Department of Neuroscience, Albert Einstein College of Medicine, Bronx, New York, USA; 4 Department of Neurology, Albert Einstein College of Medicine, Bronx, New York, USA; 5 Department of Medicine, Albert Einstein College of Medicine, Bronx, New York, USA

**Keywords:** Lowe syndrome, Dent disease, EFEMP1, Glaucoma, Macular degeneration, Cataracts, OCRL, MEIS2, TMEM132, SPON1, DPP10, Kv4.2

## Abstract

**Background:**

Lowe syndrome (LS) is caused by loss-of-function mutations in the X-linked gene OCRL, which codes for an inositol polyphosphate 5-phosphatase that plays a key role in endosome recycling, clathrin-coated pit formation, and actin polymerization. It is characterized by congenital cataracts, intellectual and developmental disability, and renal proximal tubular dysfunction. Patients are also at high risk for developing glaucoma and seizures. We recently developed induced pluripotent stem cell (iPSC) lines from three patients with LS who have hypomorphic variants affecting the 3′ end of the gene, and their neurotypical brothers to serve as controls.

**Methods:**

In this study, we used RNA sequencing (RNA-seq) to obtain transcriptome profiles in LS and control neural progenitor cells (NPCs).

**Results:**

In a comparison of the patient and control NPCs (*n* = 3), we found 16 differentially expressed genes (DEGs) at the multiple test adjusted *p* value (padj) < 0.1, with nine at padj < 0.05. Using nominal *p* value < 0.05, 319 DEGs were detected. The relatively small number of DEGs could be due to the fact that OCRL is not a transcription factor per se, although it could have secondary effects on gene expression through several different mechanisms. Although the number of DEGs passing multiple test correction was small, those that were found are quite consistent with some of the known molecular effects of OCRL protein, and the clinical manifestations of LS. Furthermore, using gene set enrichment analysis (GSEA), we found that genes increased expression in the patient NPCs showed enrichments of several gene ontology (GO) terms (false discovery rate < 0.25): telencephalon development, pallium development, NPC proliferation, and cortex development, which are consistent with a condition characterized by intellectual disabilities and psychiatric manifestations. In addition, a significant enrichment among the nominal DEGs for genes implicated in autism spectrum disorder (ASD) was found (e.g., AFF2, DNER, DPP6, DPP10, RELN, CACNA1C), as well as several that are strong candidate genes for the development of eye problems found in LS, including glaucoma. The most notable example is EFEMP1, a well-known candidate gene for glaucoma and other eye pathologies.

**Conclusion:**

Overall, the RNA-seq findings present several candidate genes that could help explain the underlying basis for the neurodevelopmental and eye problems seen in boys with LS.

## Introduction

Lowe syndrome (LS) (OMIM #300535) is a rare genetic disorder (~ 1/500,000 males) caused by mutations in the X-linked gene, *OCRL* (*OCRL*-*1*) [1–4]. It is characterized by the triad of congenital cataracts, intellectual and developmental disability (IDD), and renal proximal tubular dysfunction [1, 5–10]. Hypotonia, epilepsy, stereotypical behaviors, and a high rate of glaucoma are also observed.

*OCRL* codes for a 901 amino acid protein, inositol polyphosphate 5-phosphatase that plays a key role in endosome trafficking, clathrin-coated pit formation, and actin polymerization, by catalyzing the removal of the 5′ phosphate from phosphatidylinositol 4,5-bisphosphate (PI(4,5)P2), phosphatidylinositol 1,4,5-trisphosphate, and inositol 1,3,4,5-tetrakisphosphate [10–16].

The molecular basis of LS has primarily been studied in fibroblasts derived from patients and immortalized cell lines (e.g., HeLa; Cos-7 cells). These studies show that abnormalities in endosome recycling, in particular, megalin receptor recycling in the proximal tubules, and primary cilia dysfunction in the eye, underlie some of the clinical features [7, 11, 17–21]. However, the neurodevelopmental and behavioral aspects of LS have not been adequately investigated in human neuronal cells or animal models. A zebrafish *ocrl1* deficiency model has been developed, in which an increase in the susceptibility to heat-induced seizures, cystic brain lesions, and reduced Akt signaling have been observed [22]. Unfortunately, *Ocrl* knockout (KO) mice have significant limitations as a model system to study the neurodevelopmental aspect of LS. The original KO mouse is asymptomatic, due to compensation by the *Ocrl* paralog, *Inpp5b*, since a double *Ocrl*/*Inpp5b* KO is embryonic lethal [23–25]. Recently, a mouse model was developed by expressing the human *INPP5B* gene, which rescues the double KO, embryonic lethal phenotype KO [26]. These mice show an endolysosomal deficit in cultured proximal tubule cells. However, learning and behavioral deficits and eye pathologies were not observed.

Because of the dearth of neurodevelopmental findings in *Ocrl* KO mice, we developed an induced pluripotent stem cell (iPSC) model from three patients and their typically developing brothers. All three LS subjects have hypomorphic mutations affecting the C-terminal end of the protein [27]. We previously showed that neural progenitor cells (NPCs) derived from patient-specific iPSCs are deficient in their capacity to produce filamentous actin fibers (F-actin) and WAVE-1, a component of the WAVE regulatory complex (WRC) that controls actin polymerization [27]. The effect of these deficits on neuronal function is currently under investigation.

Although our preliminary studies have focused on some of the known effects of OCRL described in non-neuronal cells, we are also interested in identifying molecular and cellular pathways that might be uniquely affected in neural cells. One effective approach to examine molecular disruptions in an unbiased, genome-wide manner is RNA-seq. Our previous RNA-seq studies have resulted in the discoveries of novel pathways involved in *CHD8*-associated autism spectrum disorder (ASD), schizophrenia (SZ) associated with 22q11.2 deletion syndrome, and a mouse model for Rett syndrome [28–30]. RNA-seq has also been used successfully by other groups to identify pathways of interest in neuropsychiatric and neurodevelopmental disorders [31–35].

Consequently, RNA-seq was used to screen NPCs derived from LS-specific iPSCs and controls; their typically developing brothers. At the significant level of nominal *p* value < 0.05, 319 differentially expressed genes (DEGs) were found. Among them, 16 remained statistically significant after multiple test correction at the adjusted *p* (padj) < 0.1, and nine at the padj < 0.05. However, among these, there were several strong candidates for the eye and behavioral/neurological pathologies seen in LS; most notably, *EFEMP1*, *DPP10*, and *SPON1*.

## Methods

### Subjects

The study and consent forms were approved by the Albert Einstein College of Medicine (AECOM) internal review board (IRB). A diagnosis of LS was made during infancy in each patient based on clinical findings (congenital cataracts, hypotonia), fibroblast OCRL enzyme activity, and ultimately by genotyping. The patients (LS100, LS300, LS500) harbor mutations in the 3′ end of the gene that codes for the ASH-RhoGap domain (Table 1). A detailed molecular genetic analysis of the effects of these mutations on splicing can be found in our previous publication [27]. Their neurotypically developing brothers (LS200, LS400, and LS600, respectively) served as controls. All subjects were between 11 and 25 years of age when recruited, and the sibling pairs were 2–4 years apart in age.

**Table 1:** *OCRL* mutations in patients

Patient	Exon/intron	Mutation	Genomic position (hg19)	Type
LS100	Intron 23	c.2582-1 G>T	chrX:128724122	Splice acceptor
LS300	Intron 22	c.2470-2 A>G	chrX:128723820	Splice acceptor
LS500	Exon 20	c.2179delC	chrX:128721069	Del/frame shift

### Development of iPSCs cells from peripheral blood CD34^+^ cells

iPSC lines were generated from human peripheral blood CD34^+^ cells with a CytoTune-iPS 2.0 Sendai Reprogramming Kit (Invitrogen) as previously described [36]. The growth and maintenance of the iPSCs used in this study are described in our recent publication [27].

### Generating neural progenitor cells from iPSCs using dual SMAD inhibition

A monolayer neural progenitor cell (NPC) culture protocol was adapted from the STEMCELL Technologies STEMdiff^TM^ SMADi Neural Induction Kit with slight modifications. Briefly, iPSCs were maintained in mTeSR1 with daily feeding until cells reached the point of passaging. At the start of induction, differentiated cells, if present, were manually removed and the iPSCs were washed with PBS. Gentle dissociation reagent (STEMCELL Tech) was added for 8–10 min at 37 °C. Cells were dislodged by pipetting with a sterile 1 ml pipet tip and collected in a 15 ml tube. Cell culture plates were rinsed with DMEM/F12 and added to the tube containing the cell suspension. Viable cells were counted with a hemocytometer using the Trypan Blue exclusion method. Cells were then centrifuged at 300×*g* for 5 min. Supernatant was carefully aspirated and the cell pellet was re-suspended in STEMdiff^TM^ SMADi Neural Induction Medium + 10 μM Y-27632 to obtain a final concentration of 10^6^ cell/ml. Two milliliters of cell suspension were aliquoted to one well of a 6-well plate that was pre-coated with matrigel. This was designated as passage 0 (P0). Cells were allowed to grow with daily feeding for 6 days in STEMdiff SMADi Neural Induction medium. Note that Y-27632 is not required for the daily medium changes. NPCs were ready for passage when cultures were approximately 90% confluent (6 days). For passaging, NPCs were washed with DMEM/F12 and 1 ml of accutase was added to each well for 5 min at 37 °C. Cells were dislodged with a sterile 1 ml pipet tip and collected in a 15 ml tube containing DMEM/F12. Viable cells were counted using Trypan Blue exclusion. Cells were then centrifuged at 300×*g* for 5 min. Supernatant was carefully aspirated and the cell pellet was re-suspended in STEMdiff^TM^ SMADi Neural Induction Medium + 10 μM Y-27632. Cells were plated at a density of 1.5 × 10^6^ live cells/well in a 6-well plate pre-coated with PORN/Laminin. NPCs were fed daily, without Y-27632m, and were ready for downstream applications at passage 3. At this stage, none of the cells stained for the stem cell marker, OCT4 (POU5F1) and virtually 100% were positive for the NPC markers vimentin and SOX2 (Additional file 1: Figure S1). Furthermore, we compared our NPC RNA-seq data with gene expression in multiple neural and non-neural cell lines (or tissues) analyzed by RNA-seq in the ENCODE project [37] and found that the most correlated cell line was neural progenitor cell (ENCFF663ARH, Pearson’s correlation coefficients (*r*) = 0.88) (Additional file 2: Figure S2).

### RNA-seq

Total cellular RNA was extracted using the miRNeasy Mini Kit (QIAGEN, catalogue# 217004) according to the manufacturer’s instructions (QIAGEN). An additional treatment with DNase I (QIAGEN, Valencia, CA) was included to remove genomic DNA. After passing quality control, high throughput sequencing libraries were prepared by Novogene, and 150 bp paired-end RNA-seq reads were obtained. RNA-seq reads were aligned to the human reference genome (hg19) by the software HISAT2 (v2.0.4) [38]. HTseq (v0.11.0) [39] was used to determine the read counts while the StringTie (v1.2.2) [40] was used to compute fragments per kilobase of exon per million fragments mapped (FPKM) and transcript per million (TPM) for each of the genes annotated in the GENCODE database (v29) [41], including protein-coding, non-coding, and all other transcript types. Genes with TPM > 1 in at least one of the 12 samples were used for downstream analysis. RNA-seq read counts of the two biological replicates (A/B) were merged using the “collapseReplicates” function in the software DESeq2 [42], resulting in three samples each for the patient and control NPCs for differential expression analysis genes by DESeq2. The overlap of DEGs with various disease gene lists was evaluated for statistical significance by Fisher’s test. We also performed gene set enrichment analysis (GSEA; v4.0.1), using the gene sets in the gene ontology (GO) “Biological Process” category and ranking genes (*n* = 20,728) by fold changes (“log2_Ratio_of_Classes”), and otherwise default parameters.

### Quantitative real time PCR

Quantitative real-time PCR (qPCR) was carried out on reverse-transcribed PCR using the 2^-ΔΔCt^ method as we previously described [28, 29, 43].

### Immunocytochemistry

Immunocytochemistry (ICC) was carried out as previously described [27] using mouse anti-Vimentin (Invitrogen cat#18-0052) at a 1:100 dilution, and Anti-Sox2 (StemCell Technologies cat#60055.1) at a 1:50 dilution.

### Western blotting

Proteins were prepared with ProteoExtract Complete Mammalian Proteome Extraction Kit (Millipore cat# 539779) according to the manufacturer’s protocol. Protein concentrations were verified using the Bradford method. Briefly, 30–60 μg of protein were denatured with the addition of Laemmli buffer and 2-mercaptoethanol, and boiled for 5 min. Samples were loaded onto a 12% precast polyacrylamide gel (BIO-RAD cat#456-1044). Gel electrophoresis was set at constant voltage (50 V) for the first 30 min and 120 V for the remainder of the run. The running buffer was in 1× TrisGlycine/SDS buffer. After separation by electrophoresis, proteins were transferred using the Trans-Blot® TurboTM Transfer System according to the manufacturer’s instructions. A 7-min transfer was executed using the turbo program setting. After transfer, membranes were blocked in 5% milk with gentle agitation for 1 h at room temperature. Membranes were then incubated overnight with gentle agitation at 4 °C with primary antibodies for 48 h (Anti-OCRL; Proteintech Group, catalog# 17695-1-AP, 1:500 dilution: Anti-GAPDH; ThermoFisher Scientific, catalog# MA5-15738, 1:2,000 dilution). Following primary antibody incubation, membranes were washed three times with gentle agitation in 1× TBS/T buffer (20 mM Tris Base, 0.136 M NaCl, 0.1% Tween-20). Membranes were then incubated with a secondary antibody (1:5,000 dilution) plus anti-biotin (1:2,000 dilution) for 1 h at room temperature with gentle agitation. Membranes were washed again, as above, and subsequently incubated with SuperSignal^TM^ West Dura Extended Duration Substrate (Thermo Scientific cat# 34075) for 5 min at room temperature with gentle agitation. Immediately thereafter, membranes were exposed to blue autoradiograph film for visualization. For quantification, autoradiograms were scanned and the protein of interest was normalized against a control protein, GAPDH

### OCRL knockdown

An immortalized human retinal pigmented epithelium cell line, RPE-1 (ATCC® Number: CRL-4000), was used for OCRL knockdown (KD) experiments. These were carried out with Dharmacon™ siGENOME Human OCRL siRNA (cat# D-010026-01-0005, Horizon Discovery) and were transfected into RPE cells using DharmaFECT 1 Transfection reagent (Horizon Discovery) in DMEM medium containing 10% FBS without antibiotics. siGENOME non-targeting siRNA #3 (cat#D-001210-03-05, Horizon Discovery) was used as a control. siRNAs were transfected into RPE-1 cells at a concentration of 25 nM for 72 h according to the manufacturer’s protocol.

### PCR primers used in this study

**Table:** 

Gene	Left	Right
β2M	gctcgcgctactctctcttt	caatgtcggatggatgaaac
OCRL_LS500	cctgcatgaccagaatttga	ttaaaagcgctatgctgacg
OCRLexp-F	acaggtcctgcttcccacta	tggaggtggatgtctaggca
OCRLexp2-F	atccacctccagagcaacac	gctgtgggaaggagcaatag
OCRL_KO	agagctgccctcatttcctt	tgggcctggacttgataaaa
LS100cDNA	ttttcttggaagccctgcca	tgccataaggttgggtggag
LS300cDNA	agcgtcaatgccaacatgatc	aaggagggattaggaaacgctc
OCRL_LS100/300	attgtgttggccatgaggag	ggaggcctcaggagaagact

### Sequencing primers

**Table:** 

LS100seq	aatactcttagtgcattgtatc
LS300seq	tagaagttagacagatgaaatg
LS500	cctgcatgaccagaatttga

### qPCR primers

**Table:** 

TMEM132C	cacctctatggcagctctcc	cccgactgttcttcaccact
TMEM132D	gaatcctgccagaaatccaa	gtgttggggttagcatcgtt
INPP5F	tagcgttcatgctccttcag	atatgtgtacgtcgccagca
MEIS2	ccaggggactacgtttctca	tgagtagggtgtggggtcat
SLC1A3a	gccttgagcaagtcccatct	caggatgtctgggctggaag
ADCY2	gtgcgtgctgtctgtcctat	acgatctgggcacacatcag
TMEM47	tcctttgcgctgacaaggat	tcaagggctcactcaagcaa
EFEMP1	aagtgcaatgcttgtgctcg	gcggaaggtccctatactgc
RPLPO	ccaccacagctgctcctg	ggctaagttggttgctttttgg
DPP10	gtgtttcgctgcacctatga	agggagggaacaacacacac
SPON1	aggagtagtgtcagccacct	atggttgcctctccatgtgg
NLRP2	atgctagactgggcagagga	cagtccctgaagaccagctc
CALB1	tatccaggatgtgtggctca	tgggtgtactgactggccta

## Results

### RNA-seq and bioinformatics

iPSCs from three subjects with LS and controls were differentiated into NPCs. RNA was extracted and analyzed by paired-end RNA-seq. Two independent NPC samples differentiated from each iPSC line were prepared for duplicated RNA libraries sent for RNA-seq, resulting in a total of 12 biological samples. The overall quality of the RNA-seq reads and alignment was excellent, with a range of 22,150,351–29,297,502 reads, and alignment rates from 85.58 to 90.67% (Additional file 3: Table S1). In addition, we were able to confirm the mutant *OCRL* genotype for each patient sample in the RNA-seq reads: loss of intron 23/exon 24 splice site with cryptic splice site in exon 24 (LS100); loss of intron 22/exon 23 splice site with absence of exon 23 in the final transcript (LS300); and a “C” del in exon 20 (LS500) (Table 1; Additional file 4: Figure S3A-C).

**Fig. 1:**
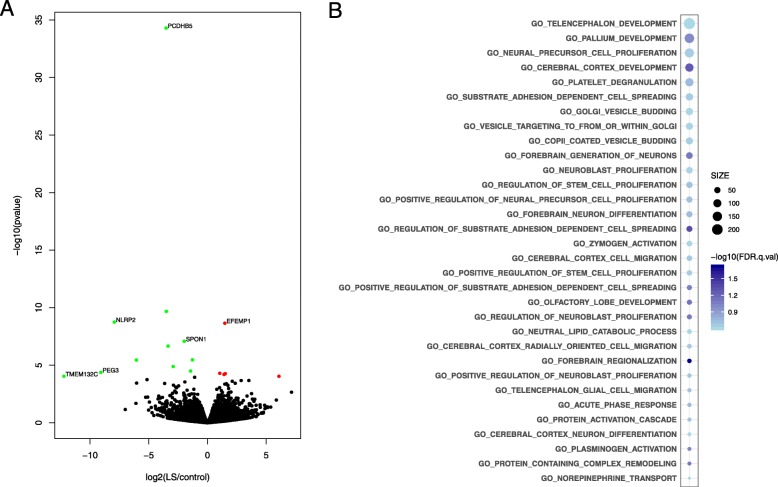
**a** Volcano plot showing differentially expressed genes (DEGs) at padj < 0.1; lower expression in LS NPCs compared with controls (green); higher expression (red). **b** Gene ontology terms enriched in genes that are up-regulated in LS NPCs, as determined by gene set enrichment analysis. The size of the circles corresponds to the number of DEGs in a GO term, and the color intensity corresponds to the –log10 (FDR) of enrichment significance. Plots are all GO terms with FDR < 25%

Since the gene expression profiles of the two duplicated samples were highly correlated, we merged them for determining DEGs in NPCs comparing LS samples and their sibling controls (*n* = 3) using the software DESeq2 [42]. At a nominally significant level (*p* < 0.05), 319 DEGs were found (164 genes expressed at higher levels in LS; 155 lower) (Additional file 5: Table S2). At the multiple test adjusted *p* value (padj) < 0.05, we found nine DEGs (*PCDHB5*, *BARHL1*, *NLRP2*, *EFEMP1*, *SPON1*, *GDA*, *CALB1*, *ZNF736*, *PITX2*), with an additional seven at padj between 0.05 and 0.1 (*INPP5F*, *PEG3*, *SPINK5*, *DPP10*, *MAFB*, *OTP*, *TMEM132C*) (Fig 1a). Of the 16 DEGs that achieved padj < 0.1, five are known SZ, ASD, IDD or eye disorder candidate genes (*DPP10*, *GDA*, *PITX2*, *EFEMP1*, *SPON1*) (Table 2).

**Table 2:** Genes involved in ASD, SZ, ID, and eye disorders that overlap with the 319 nominal DEGs. Two additional genes were included based on literature support: *MEIS1* is a glaucoma candidate that is not in the NEI/NIH eye database, and *SPON1* is not in any of the SZ candidate gene databases, but was recently identified in an exome sequencing study (both denoted by asterisk *). Genes shown in bold type are the DEGs at padj < 0.1

ASD	SZ	IDD	EYE
*AFF2*	*BMP6*	*AFF2*	*CNGB1*
*ANO5*	*CA8*	*CA8*	*COL2A1*
*CA8*	*CACNA1C*	*EEF1A2*	***EFEMP1***
*CACNA1C*	*CHN2*	*MLC1*	MEIS2*
*CADPS2*	*CHRFAM7A*	*RELN*	***PITX2***
*COMT*	*COMT*	*TH*	SPON1*
*DNAH3*	*MLC1*	*XYLT1*	*TNFRSF11B*
*DNER*	*PCDHA6*		
***DPP10***	*PNPO*		
*DPP6*	*PPP1R16B*		
*EEF1A2*	*PROZ*		
*GABRG3*	*RELN*		
*GALNT10*	*SLC1A3*		
***GDA***	*SNCG*		
*KCND3*	SPON1*		
*KCNJ12*	*TH*		
*MEIS2*	*ADCY2*		
*OTX1*			
*PCDHA6*			
RELN			
RORA			
SYT17			
ZNF385B			

To address the potential limitation in using thresholds for selecting DEGs, we also applied the software GSEA to find enriched GO terms [44, 45]. For genes expressed higher in patient NPCs, 32 “Biological Process”-related GO terms were enriched at false discovery rate (FDR) < 25% (Fig. 1b). The top GO terms for LS upregulated genes were telencephalon development, pallium development, NPC proliferation, and cortex development, which are consistent with a condition characterized by intellectual disabilities and psychiatric manifestations. For genes showing decreased expression in LS NPCs (an increase in controls), no GO terms passed the 25% FDR threshold. At a nominal *p* value < 0.01, the top terms were vasculature development, nuclear-transcribed mRNA catabolic process, and protein kinase B signaling (Additional file 6: Figure S4).

**Fig. 2:**
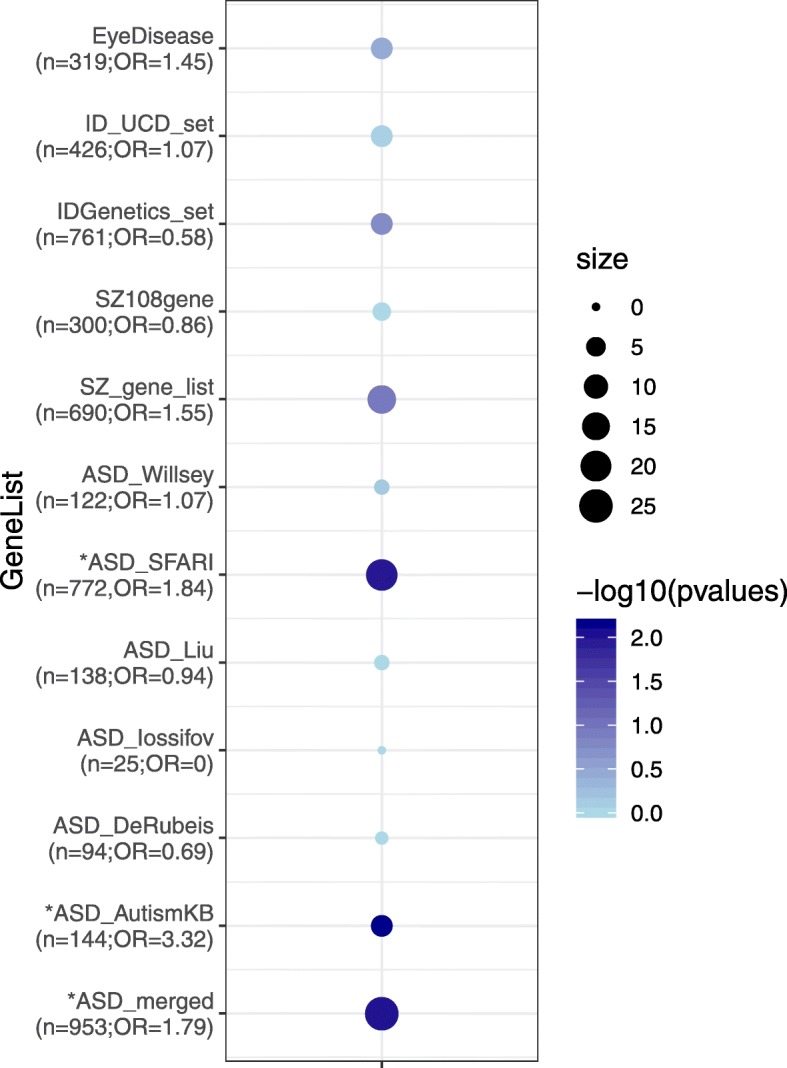
Overlap of 319 nominal DEGs with genes implicated in autism spectrum disorders (ASD), schizophrenia (SZ), intellectual disability (ID), and eye disease. See main text for references and web sites. The size of the circles corresponds to the number of DEGs that overlap with disease-associated genes in the various data sets, while the color intensity corresponds to the –log10 (*p* value) from Fisher’s test. The *n* is the number of genes expressed in our NPC samples; OR is odds ratio; * denotes *p* < 0.05

Finally, we analyzed the overlap of the 319 nominal DEGs with lists of genes that were associated with ASD, schizophrenia (SZ), IDD, and eye diseases in previous studies [46–53] (SFARI [https://gene.sfari.org/autdb/GS_Home.do]; https://neibank.nei.nih.gov/cgi-bin/eyeDiseaseGenes.cgi) (Additional file 7: Table S3). We found that ASD-related genes showed a significant overlap with our DEGs (Fig. 2). Among the more interesting DEGs in the context of behavioral and cognitive problems are *AFF2*, *ADCY2*, *DNER*, *DPP10*, *CACNA1C*, *MEIS2*, *GDA*, *RELN*, which have been linked to neurodevelopmental disorders and neuropsychiatric disorders in multiple studies (Table 2) [54–81]. There was also overlap for genes involved in intellectual disabilities, including *AFF2*, *AIFM1*, *CA8*, *EEF1A2*, *MLC1*, and *XYLT1* [82–87], but statistically the overlap is not significant.

Interestingly, some enrichment (OR = 1.45; Fig. 2) was also seen for DEGs involved in eye disorders, consistent with the clinical problems seen in LS. One such gene is *EFEMP1* (EGF containing fibulin extracellular matrix protein 1), which was the most significantly upregulated DEG in our study. *EFEMP1* has been implicated in glaucoma in several genome-wide association studies (GWAS), macular degeneration, age-related macular dystrophy, and Doyne honeycomb retinal dystrophy [88–94]. *EFEMP1* has also been found to be associated with suicidal behavior in genetic and molecular studies [95–98].

Two other DEGs that are involved in both neurodevelopmental and eye pathologies are *MEIS2* and *SPON1*. *MEIS2* codes for a homeobox protein, and is a key regulator of trabecular meshwork, lens, and retina development [99–102]. It was also identified as a risk factor in an open-angle glaucoma GWAS [103], and *MEIS2* missense mutations and microdeletions have been found in patients with ASD and developmental delay [60–62, 81, 104].

*SPON1* codes for SPONDIN-1, an extracellular matrix component involved in axon guidance. Mutations have been found in a SZ exome sequencing study, and genome-wide association studies have implicated the gene in the rate of cognitive decline and dementia severity in Alzheimer disease [105–108]. It is also a differentially expressed protein in the development of cataracts, and is a major target of the Pax6 pathway during lens development [109].

**Fig. 3:**
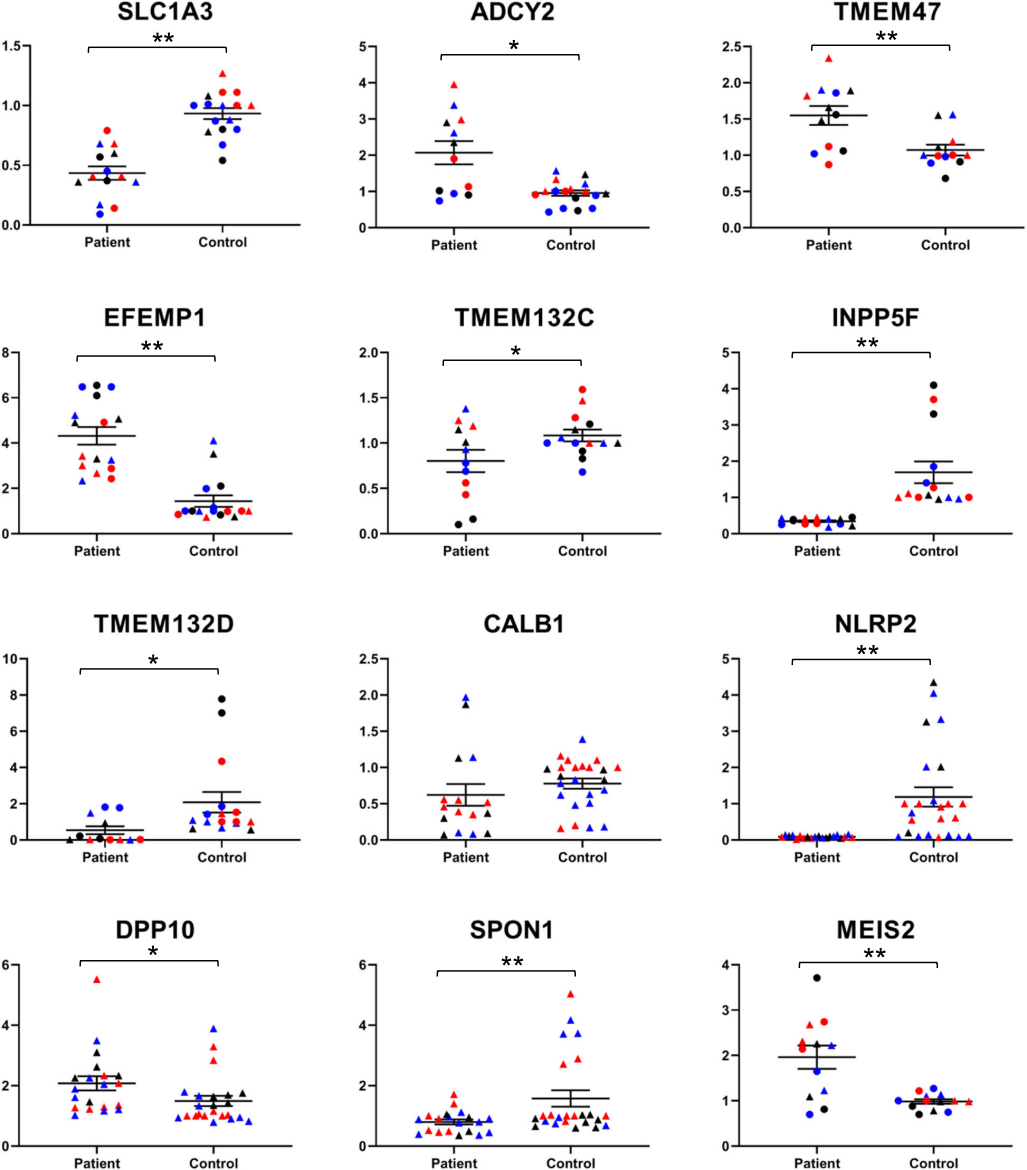
Quantitative real time PCR (qPCR). Selected up and down-regulated genes were analyzed by reverse transcribed PCR using the 2^-ΔΔCt^ method with *RPLPO* as a control gene. The *y*-axis is the relative expression compared with a common control RNA. Asterisk(s) (*) and (**) denote *p* < 0.05 and *p* < 0.01 (two-tailed student’s *t* test). The RNA samples were the same ones used in the RNA-seq analysis, combined with a third set of RNAs not used in the RNA-seq experiment. Each sample was analyzed by qPCR twice

We validated six DEGs by qPCR that were significant at padj < 0.1 (*EFEMP1*, *TMEM132C*, *INPP5F*, *DPP10*, *SPON1*, *CALB1*), as well as six others that were nominally significant (*p* < 0.05), which showed significant differences that were in agreement with the RNA-seq data, with the exception of CALB1. This showed the expected decrease, but only a trend toward statistical significance was found (*p* < 0.08) (Fig. 3). The analysis was carried out on the two original RNA samples sent for sequencing and an independent set of NPCs.

These findings suggest that the neurodevelopmental features and eye pathologies seen in LS are mediated, in part, by altered expression of the DEGs we identified.

### OCRL KD in RPE-1 cells

**Fig. 4:**
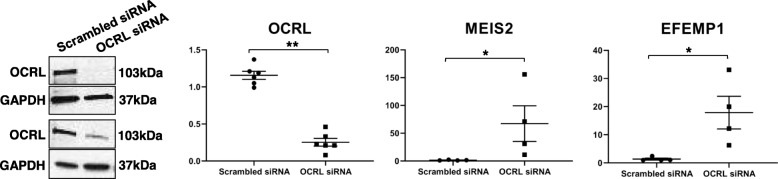
OCRL knockdown. Left panel shows a western blot of OCRL along with a control protein, GAPDH, after NPCs were exposed to an OCRL siRNA and a scrambled control. Two independent KD experiments were carried out. The right three panels are qPCR results for *OCRL, MEIS2* and *EFEMP1* carried out on two independent RNA samples, both analyzed in duplicate, as described in the methods section an in the Fig. 3 legend

To further validate the connection between OCRL expression and genes involved in eye pathology, we knocked down OCRL expression in a human retinal pigmented epithelial cell line, RPE-1. An R345W mutation in *EFEMP1* causes Doyne honeycomb retinal dystrophy [92], which leads to activation of the alternative complement pathway in RPE cells [110]. RPE-1 cells were transfected with siRNAs targeting *OCRL* mRNA, and a scrambled control. As seen in Fig. 4, significant decrease in OCRL protein (left panel), and *OCRL* mRNA (right panel) occurred in cells treated with *OCRL* siRNA compared with cells treated with the scrambled control siRNA (83% decrease in normalized OCRL protein, *p* = 0.02; 70% decrease in normalized *OCRL* mRNA, *p* = 3.1E-07, Student’s *t* test, two-tailed). This was accompanied by extremely large increases in the expression of *MEIS2*, which is a regulator of retinal development, as noted above, and *EFEMP1* mRNA (*p* = 0.04 and 0.03, respectively, Student’s *t* test, two-tailed). The relatively modest levels of statistical significance compared with large fold changes are due to the small sample size (two independent KD experiments; qPCR carried out in duplicate).

These findings show that the increase in expression of these genes seen in LS NPCs is directly related to the loss of function, patient-specific *OCRL* mutations found in our subjects.

## Discussion

Overall, the transcriptome findings were relatively modest with respect to the number of DEGs found in all three LS/control pairs compared to differences found between patients and controls in other iPSC disease-model systems that we have analyzed. For example, transcriptome analyses on NPCs, monolayer neurons, and cerebral organoids derived from *CHD8* haploinsufficient iPSC lines resulted in hundreds of shared DEGs with highly significant enrichment of pathways relevant to ASD pathogenesis [28, 30]. This could reflect the biological function of the underlying candidate genes. *CHD8* codes for a member of the CHD family of ATP-dependent chromatin-remodeling factors, so major changes in gene expression through both direct and indirect effects on target genes are expected from its haploinsufficiency. By contrast, all of the functions so far attributed to OCRL in non-neuronal cells occur at the post-translational level. As such, many of our observed DEGs could be due to secondary effects through a variety of potential OCRL-affected pathways, such as altered recycling of growth factor receptors linked to activation of transcription factors, and increases in membrane-associated phosphatidylinositol 4,5-bisphosphate (PI(4,5)P2), which can potentially affect protein kinase C-mediated gene expression. Interestingly, a transcription factor binding motif analysis of the promoters of the 319 DEGs found a nominally significant enrichment of a PITX2 motif (*p* < 0.05, vs. all promoters). The expression of *PITX2*, which is important for the development of the anterior chamber of the eye and is a glaucoma candidate gene [111–113], was significantly reduced in the LS NPCs (Additional file 5: Table S2).

Another factor that could have limited the full potential of RNA-seq to help understand disease pathogenesis in our study is that NPCs derived from iPSCs are grown in vitro under very specific conditions, as opposed to NPCs and other neuronal cells derived from a developing brain where the full repertoire of growth factors and cell-cell interactions that might affect NPC differentiation and gene expression potentially influenced by *OCRL* could come into play. Testing this hypothesis in mammalian brains will have to await the development of a suitable mouse model that recapitulates the neurodevelopmental features of LS.

**Fig. 5:**
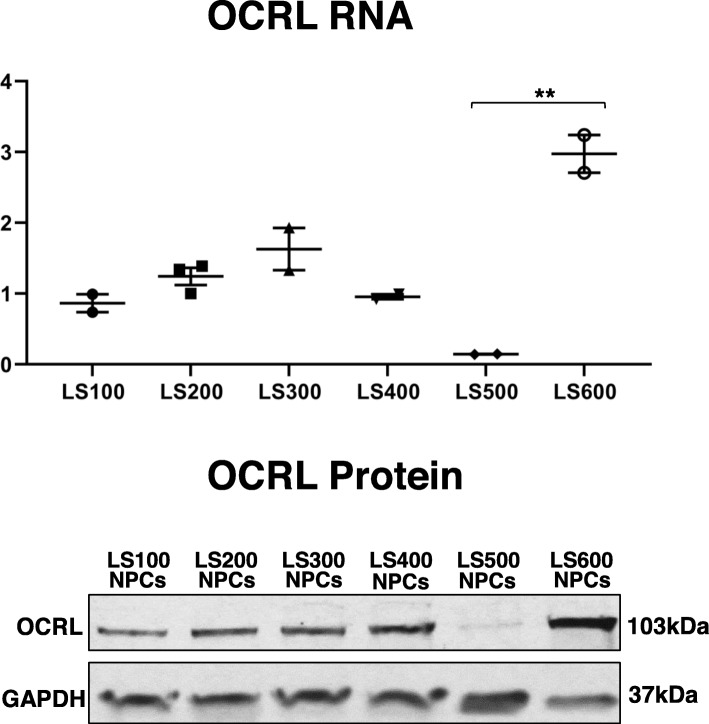
OCRL protein and RNA. OCRL RNA was analyzed by qPCR (top) as described in the Fig. 3 legend. OCRL protein (bottom) was analyzed by Western blotting, as described in the methods section. The Western blot was done twice with two independent samples

Finally, the number of DEGs might have been reduced because of differences in *OCRL* expression in the three LS NPC samples. Although all three patients have mutations in the ASH-RhoGAP binding domains that generally produce hypomorphic variants, expression of LS500 is substantially lower than his brother and the other LS/control samples (Additional file 5: Table S2). This was confirmed by qPCR (Fig. 5, top panel). The decrease in *OCRL* mRNA in LS500 is most likely due to nonsense-mediated decay, and is accompanied by a marked decrease in OCRL protein (Fig. 5, bottom panel). These observations suggest that producing a truncated, dysfunctional OCRL protein may have additional effects on gene expression and other phenotypes compared with a simple reduction in OCRL protein levels. This could help explain phenotypic differences seen in LS patients. For example, while every patient has the triad of congenital cataracts, IDD, and renal proximal tubular dysfunction, subgroups of patients have epilepsy, stereotypical behaviors, and glaucoma. In addition, this hypothesis could also help explain the more dramatic clinical differences seen in LS and patients with *OCRL*-associated DENT-2 disease, the latter of  which is characterized by renal disease, without eye and behavioral manifestations [114–117]. In addition, we previously found differences in the production of F-actin and WAVE-1 in LS NPCs compared with NPCs made from a null *OCRL* iPSC line that we generated using CRISPR-Cas9 gene editing [27]. However, in that study, the LS500/LS600 pair showed the same abnormality as the other two patient/control sets. Thus, other factors need to be invoked to help explain the clinical and molecular heterogeneity seen in LS and DENT-2 disease, such as genetic background. Generating additional patient-specific lines, as well as creating null and patient-specific mutations using CRISPR-Cas9 gene editing in isogenic lines to control for genetic background, will be needed to sort through these intriguing possibilities.

We presented our results by a combined analysis of the patient and control NPCs. We have also tried to identify DEGs between the LS samples and controls of each family. The sample size was small (*n* = 2), thus statistical power is weak, but a few thousand DEGs were detected at fold change > 2 and adjusted *p* < 0.05 between LS and their corresponding sibling NPC controls (data not shown). The overlap of the DEGs from the three families, however, was relatively small, indicating a high level of gene expression variation in these NPC samples. Additional LS/control sets, analysis of multiple clones from the same subjects, and analyzing isogenic control/CRISPR-edited lines will be needed to resolve this issue. We are currently generating such lines.

As an alternative to DESeq2, we also applied the limma voom differentiation expression analysis [118] to the jointed analysis of our samples, using its “duplicateCorrelation” function to account for two replicates per iPSC line. However, it did not yield DEGs by our statistical criteria of padj < 0.05 (data not shown).

Despite the lack of highly enriched pathways from our DEG analysis, a number of interesting candidate genes emerged that strongly suggest a role in the neurodevelopmental and eye problems associated with LS, and cellular phenotypes we previously identified in NPCs. As noted in the results section, four out of the top 16 DEGs genes are known SZ, ASD, IDD candidate genes (DPP10, GDA, PITX2, SPON1), and overall, there was a significant overlap between the 319 DEGs and ASD candidate genes (Fig. 2).

Two other DEGs of note are *TMEM132C* and *TMEM132D*, which are feasible candidates for the F-actin/WAVE1 abnormalities we previously found in LS NPCs [27]. These genes code for members of a family of transmembrane, cell-surface molecules expressed in the brain [119]. TMEM132 proteins have an intracellular WAVE regulatory complex interacting receptor sequence cytoplasmic motif, which is a key regulator of actin polymerization by initiating F-actin nucleation through an interaction with the Arp2/3 complex [120]. Whether TMEM132 proteins affect WAVE-1 expression in LS NPCs remains to be determined.

One of the more interesting findings in the NPC transcriptome analysis are the DEGs implicated in eye pathology. This could represent differential expression in eye tissue that happen to be similarly affected in neural cells, or to common developmental pathways, although this remains to be determined. On the other hand, recent research suggests that some forms of glaucoma should be viewed as neurodegenerative disorders caused by retinal and optic nerve injury [121, 122], so it is possible that the primary defect related to these DEGs is due to aberrant expression in neuronal cells.

The most significant DEGs related to LS eye pathology are *EFEMP1*, *MEIS2*, and *SPON1I*, as noted above*. EFEMP1* is particularly interesting from a therapeutic perspective because it is connected to several potentially druggable pathways, including complement activation, the EGF receptor, and BMP7 and TGFβ2 signaling [110, 123–125]. In addition, EFEMP1, as a protein secreted into the extracellular matrix, could be a target for therapeutic intervention as well.

With respect to *EFEMP1*, *MEIS2*, and *SPON1*, and glaucoma risk in LS, the fact that these genes have been implicated in glaucoma and other eye disorders suggests that understanding how they cause severe eye disease in LS could have much broader public health implications, considering the high prevalence of these conditions in the general population. Thus, the iPSC lines we have developed could be very useful for screening small molecule modulators of EFEMP1 and other eye-related DEGs for a wide range of eye disorders. Their role in eye pathology can now be analyzed in our iPSC model system since several protocols have been published for inducing differentiation into various types of eye tissues [126, 127].

## Limitations

A limitation of the study is the sample size, which is a general limiting factor for most iPSC studies, considering the expense and time it takes to cultivate these lines. In addition, there is a dearth of protein and functional validation, which means that individual DEG findings may ultimately fail to be biologically relevant. Nevertheless, our findings are still of great interest because the differentially expressed genes are consistent with what is known about LS with respect to both neurodevelopmental and eye pathologies. We plan on increasing our sample size in the next year to improve the scientific rigor of future transcriptomic studies.

## Conclusions

RNA-seq analysis of iPSC-derived NPCs from patients with Lowe syndrome and their typically developing brothers identified 319 DEGs, which are enriched with genes that have been identified as ASD candidates. In addition, several DEGs code for genes that have been implicated in the development of cataracts, glaucoma, and retinal disease. Altered expression of these genes may play a role in the behavioral and ocular problems occurring in LS and connect this extremely rare condition at a pathophysiological level to a much wider population of disorders. The study also points to several feasible targets for therapeutic intervention.

## Supplementary information

**Additional file 1: Figure S1.** Immunocytochemistry (ICC) of NPCs used in the RNA-seq study (one set) showing Vimentin and Sox2 staining (A and B, respectively) with a nuclear stain (DAPI).

**Additional file 2: Figure S2.** Pearson’s correlation coefficients of the gene expression between NPCs described in the current study and ~300 samples from the ENCODE project. Samples with coefficients > 0.85 are shown and the highest correlations are NPCs from ENCODE (ENCFF663ARH), followed by a human neuroblastoma cell line, SK-N-DZ.

**Additional file 3: Table S1.** Quality of RNA-seq Reads and Mapping. The number of reads, alignment rates, and reads across different gene regions are similar. The instrument ID, run number, lane number and flowcell ID were provided by the Novogene.

**Additional file 4: Figure S3.** A and B. Sashimi plots of RNA-seq reads confirming cryptic splice in exon 24 in LS100, and loss of exon 23 in LS200, as described in Barnes et al. C. shows deletion of "C" in exon 20 in LS500.

**Additional file 5:Table S2.** Entire gene list arranged in descending order of significance level (pval), The top 16 genes with padj < 0.1 are in bold type.

**Additional file 6: Figure S4.** Gene Set Enrichment Analysis (GSEA) for genes that are up-regulated in controls (down-regulated in LS). GO terms were selected by nominal pval < 0.01 because none passed FDR < 25%.

**Additional file 7: Table S3.** Overlap of nominal DEGs with ASD, ID, SZ and eye disease gene datasets. Citations are included in the table.

## Data Availability

RNA-seq data can be accessed at the Gene Expression Omnibus (GEO), (https://www.ncbi.nlm.nih.gov/geo/), accession number GSE129310, review token, yzuluyoyrtavdkr.

## Abbreviations

DEG: Differentially expressed gene
FPKM: Fragments per kilobase of exon per million fragments mapped
FDR: False discovery rate
GO: Gene ontology
GWAS: Genome-wide association study
iPSC: Induced pluripotent stem cell
KO: Knockout
NPC: Neural progenitor cell
PCR: Polymerase chain reaction
qPCR: Quantitative real-time PCR
Pval: *P* value
ASD: Autism spectrum disorders
SZ: Schizophrenia
BD: Bipolar disorder
ICC: Immunocytochemistry
PI(4,5)P2: Phosphatidylinositol 4,5-bisphosphate
IRB: Internal review board
