# Supplementary figures and images for: Transcriptome analysis of neural progenitor cells derived from Lowe syndrome induced pluripotent stem cells: identification of candidate genes for the neurodevelopmental and eye manifestations
Source: J Neurodev Disord. 2020 May 11;12:14. doi: 10.1186/s11689-020-09317-2 (PMC7212686; doi:10.1186/s11689-020-09317-2)

**Vimentin / DAPI**

**LS100**

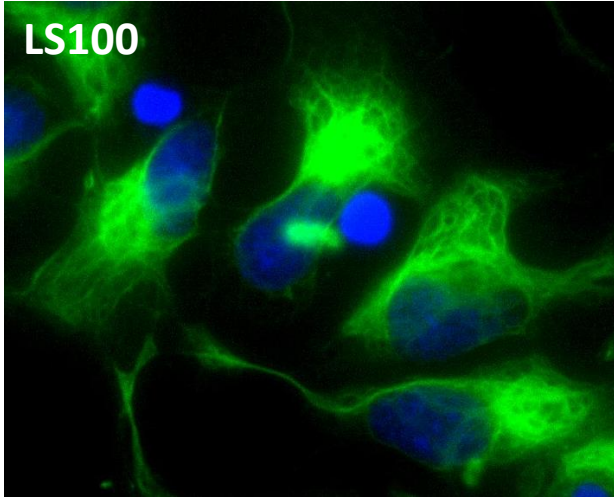

**LS300**

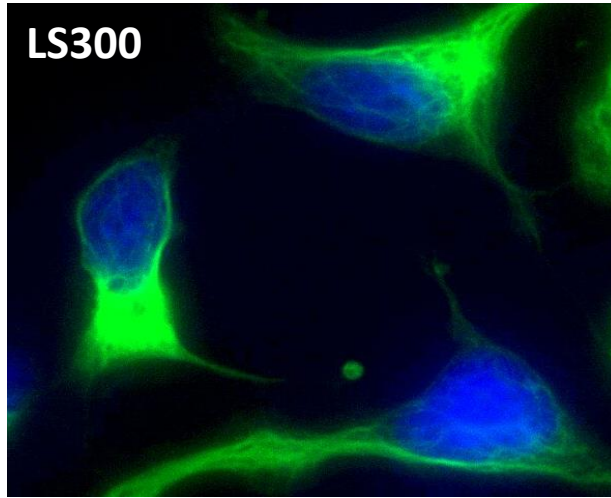

**LS500**

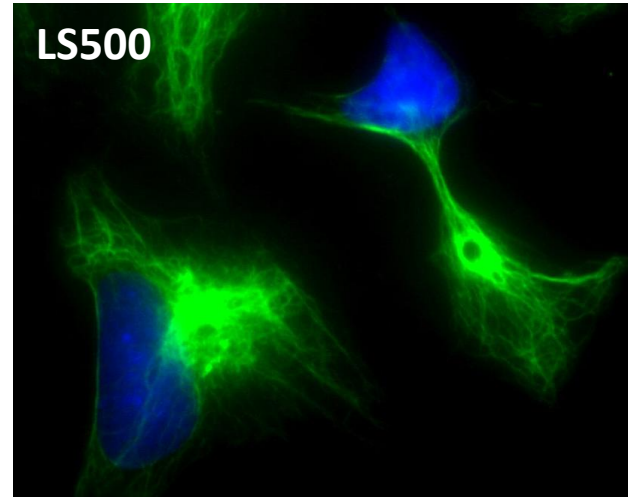

**LS200**

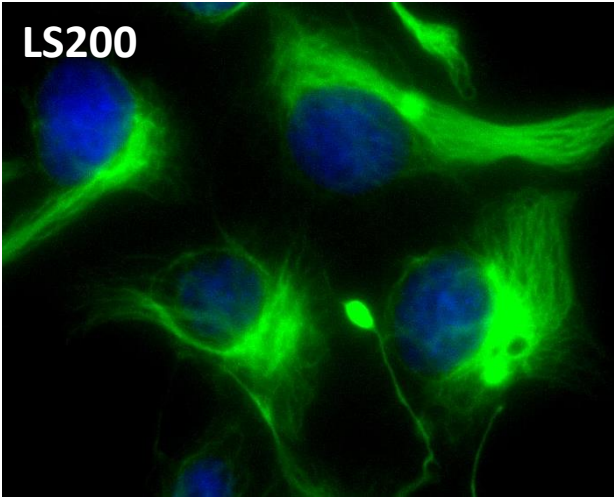

**LS400**

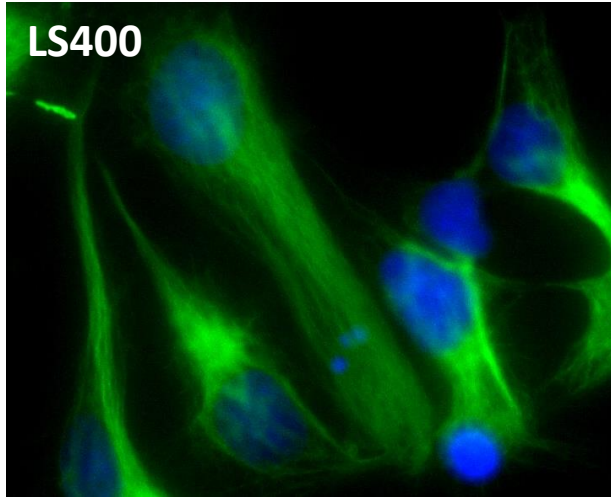

**LS600**

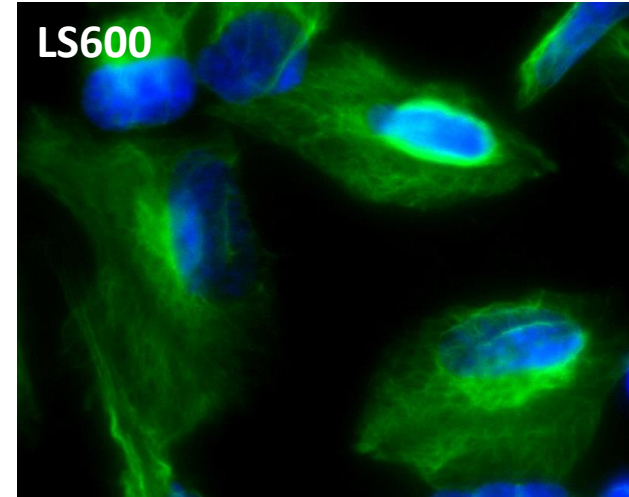

**Sox2 / DAPI**

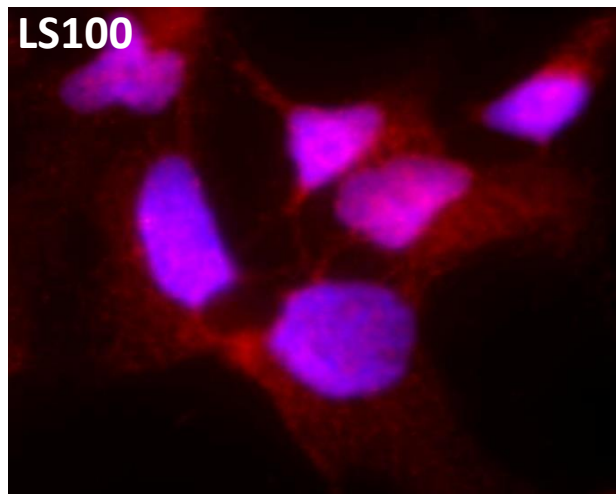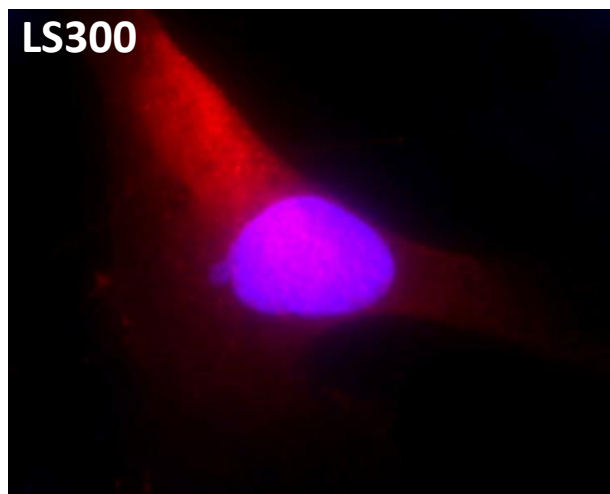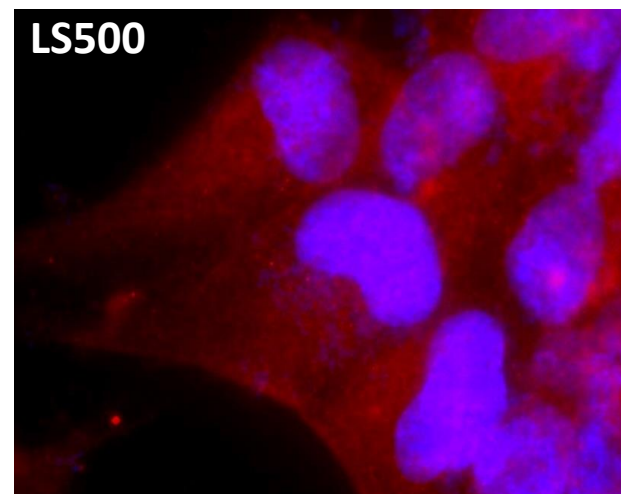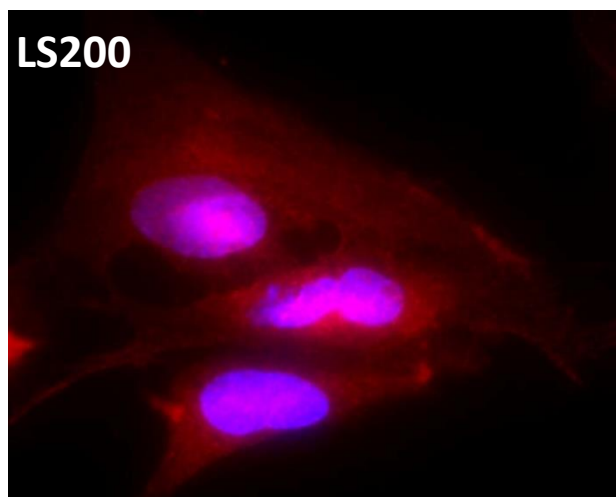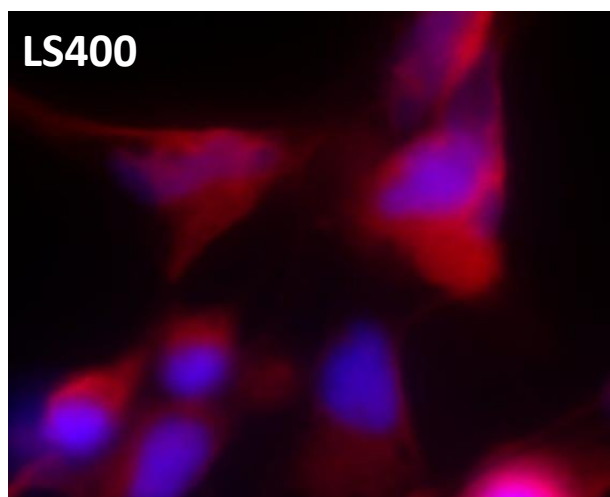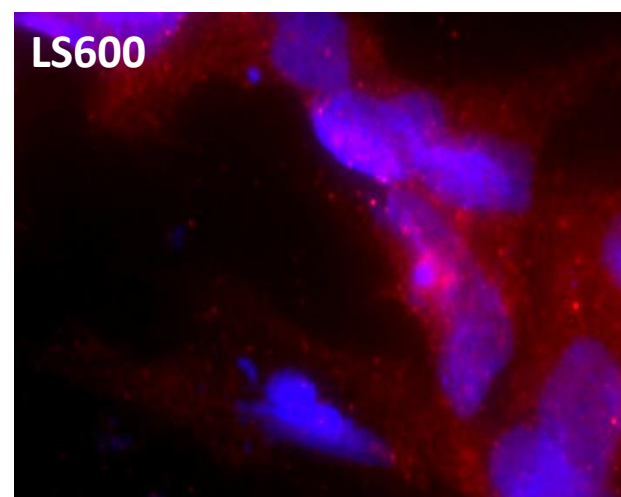

Supplement: Supplementary file 1 — Additional file 1: Figure S1. Immunocytochemistry (ICC) of NPCs used in the RNA-seq study (one set) showing Vimentin and Sox2 staining (A and B, respectively) with a nuclear stain (DAPI). [file 11689_2020_9317_MOESM1_ESM.pdf_]

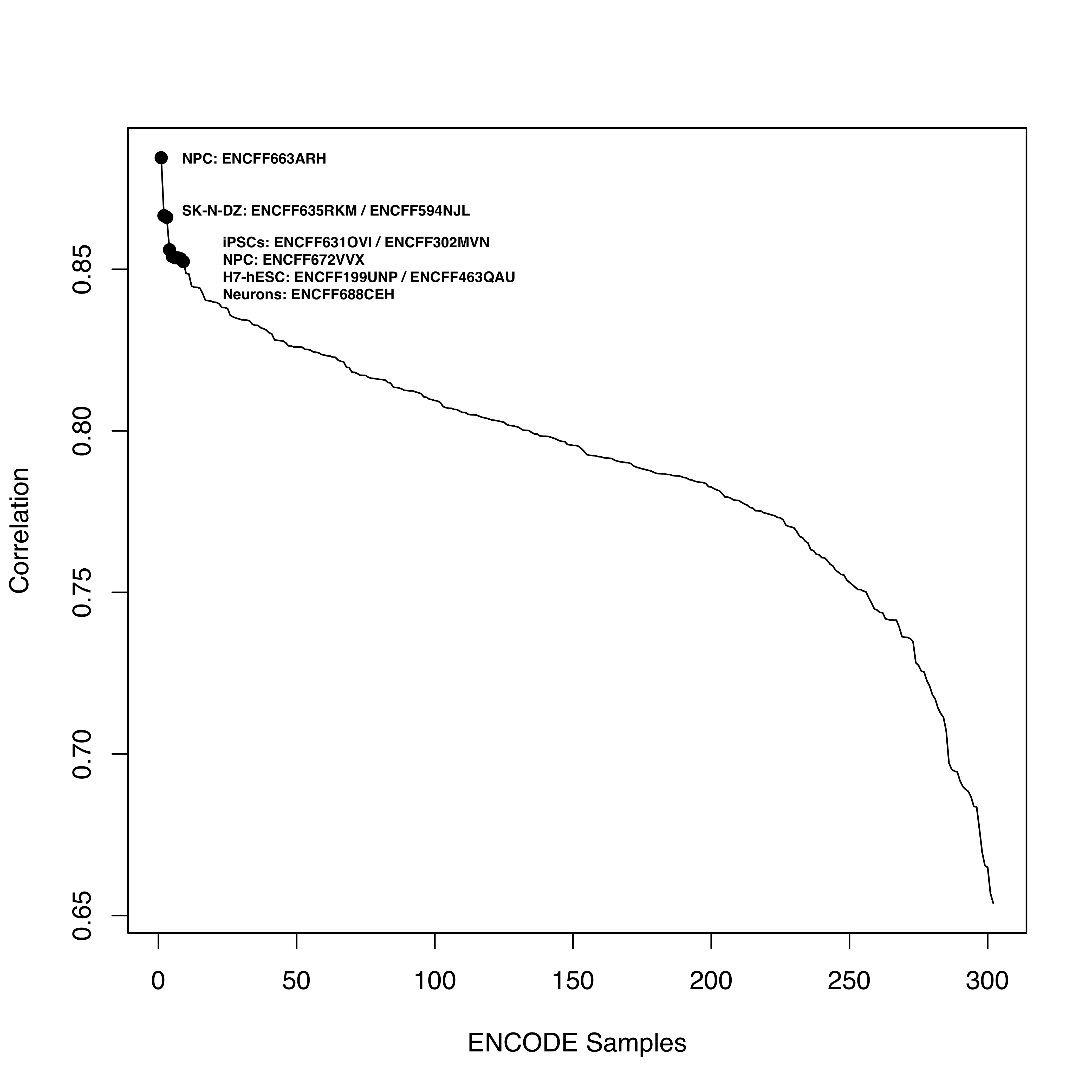

Supplement: Supplementary file 2 — Additional file 2: Figure S2. Pearson’s correlation coefficients of the gene expression between NPCs described in the current study and ~300 samples from the ENCODE project. Samples with coefficients > 0.85 are shown and the highest correlations are NPCs from ENCODE (ENCFF663ARH), followed by a human neuroblastoma cell line, SK-N-DZ. [file 11689_2020_9317_MOESM2_ESM.png]

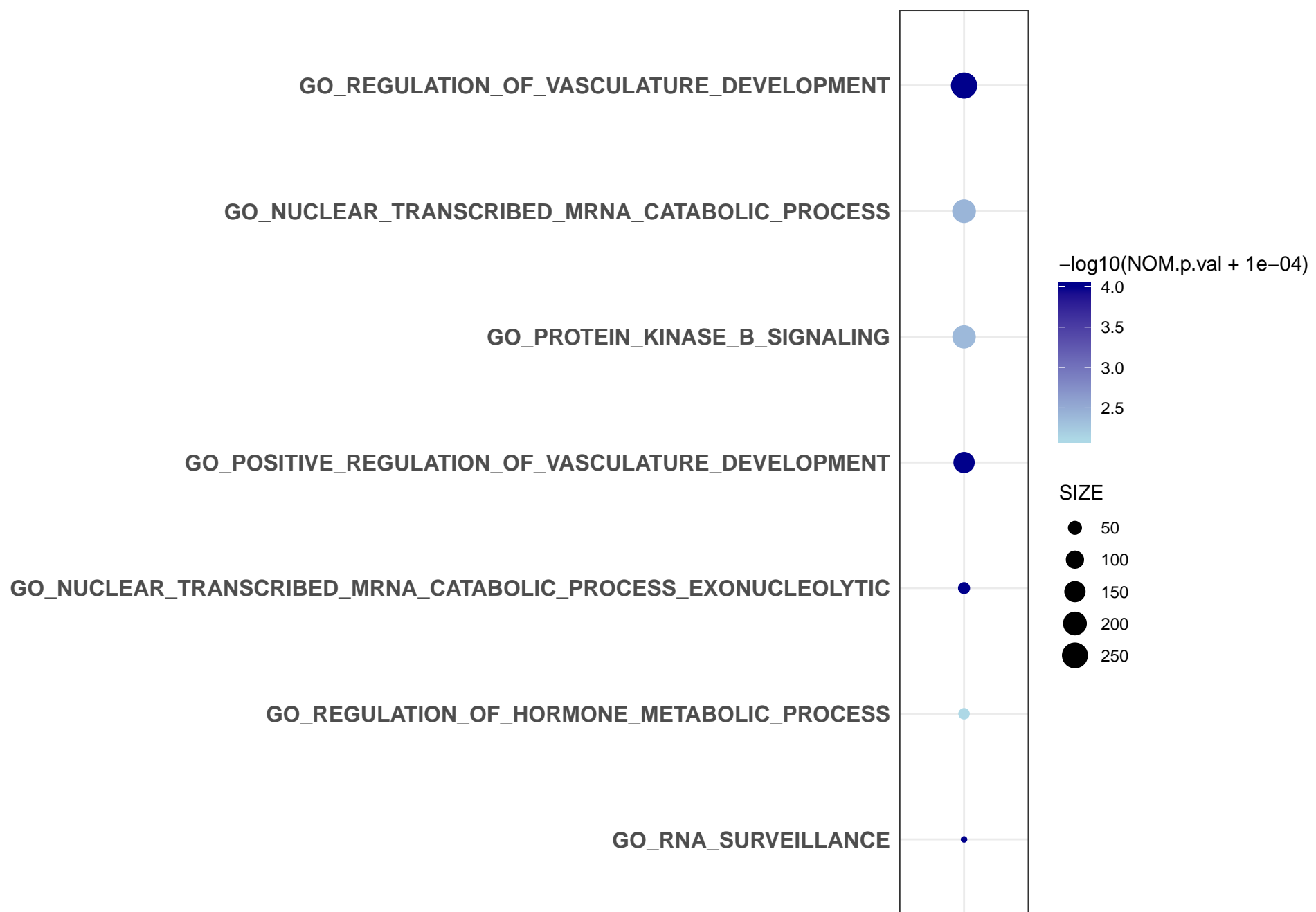

Supplement: Supplementary file 6 — Additional file 6: Figure S4. Gene Set Enrichment Analysis (GSEA) for genes that are up-regulated in controls (down-regulated in LS). GO terms were selected by nominal pval < 0.01 because none passed FDR < 25%. [file 11689_2020_9317_MOESM6_ESM.pdf]
