# Supplementary material for: Transcriptome analysis of neural progenitor cells derived from Lowe syndrome induced pluripotent stem cells: identification of candidate genes for the neurodevelopmental and eye manifestations
Source: J Neurodev Disord. 2020 May 11;12:14. doi: 10.1186/s11689-020-09317-2 (PMC7212686; doi:10.1186/s11689-020-09317-2)
Supplement: Supplementary file 3 — Additional file 3: Table S1. Quality of RNA-seq Reads and Mapping. The number of reads, alignment rates, and reads across different gene regions are similar. The instrument ID, run number, lane number and flowcell ID were provided by the Novogene. [file 11689_2020_9317_MOESM3_ESM.pdf]

Additional file 3: Table S1
Quality of Reads and Mapping

Samples	Instrument ID	Run Number	Flowcell ID	Lane Number	Reads	Alignment Rate	coding_bases %	utr_bases %	intronic_bases %	intergenic_bases %
LS100A	A00564	55	HFJ52LSXX	3	24594950	89.11%	51.14%	32.07%	8.37%	8.42%
LS100B	A00564	55	HFJ52LSXX	3	29297502	90.52%	53.37%	30.46%	7.43%	8.73%
LS200A	A00261	116	HGVHLLSXX	1	23361572	89.21%	51.96%	29.46%	10.28%	8.29%
LS200B	A00261	111	HFJ5KLSXX	3	22150351	86.91%	52.23%	28.86%	10.53%	8.38%
LS300A	A00197	83	HHFY7DMXX	1	23631444	86.15%	53.48%	28.73%	9.57%	8.22%
LS300B	A00197	83	HHFY7DMXX	1	22929806	85.78%	53.30%	28.38%	10.28%	8.04%
LS400A	A00197	83	HHFY7DMXX	1	23375334	86.37%	55.14%	28.23%	8.53%	8.09%
LS400B	A00261	116	HGVHLLSXX	1	23262554	89.06%	55.26%	28.35%	8.67%	7.72%
LS500A	A00261	116	HGVHLLSXX	1	22361193	85.97%	54.39%	26.89%	11.14%	7.58%
LS500B	A00197	83	HHFY7DMXX	1	23307319	87.86%	50.10%	29.71%	11.46%	8.73%
LS600A	A00197	83	HHFY7DMXX	1	23787697	85.58%	52.95%	28.07%	9.91%	9.07%
LS600B	A00197	83	HHFY7DMXX	1	24604823	87.64%	51.34%	29.55%	10.56%	8.56%
