# Supplementary material for: Transcriptome analysis of neural progenitor cells derived from Lowe syndrome induced pluripotent stem cells: identification of candidate genes for the neurodevelopmental and eye manifestations
Source: J Neurodev Disord. 2020 May 11;12:14. doi: 10.1186/s11689-020-09317-2 (PMC7212686; doi:10.1186/s11689-020-09317-2)

Additional file 4: Figure S3A

Sashimi plot of *OCRL* gene showing exons 23 and 24 in LS100 (red) and LS200 (blue)

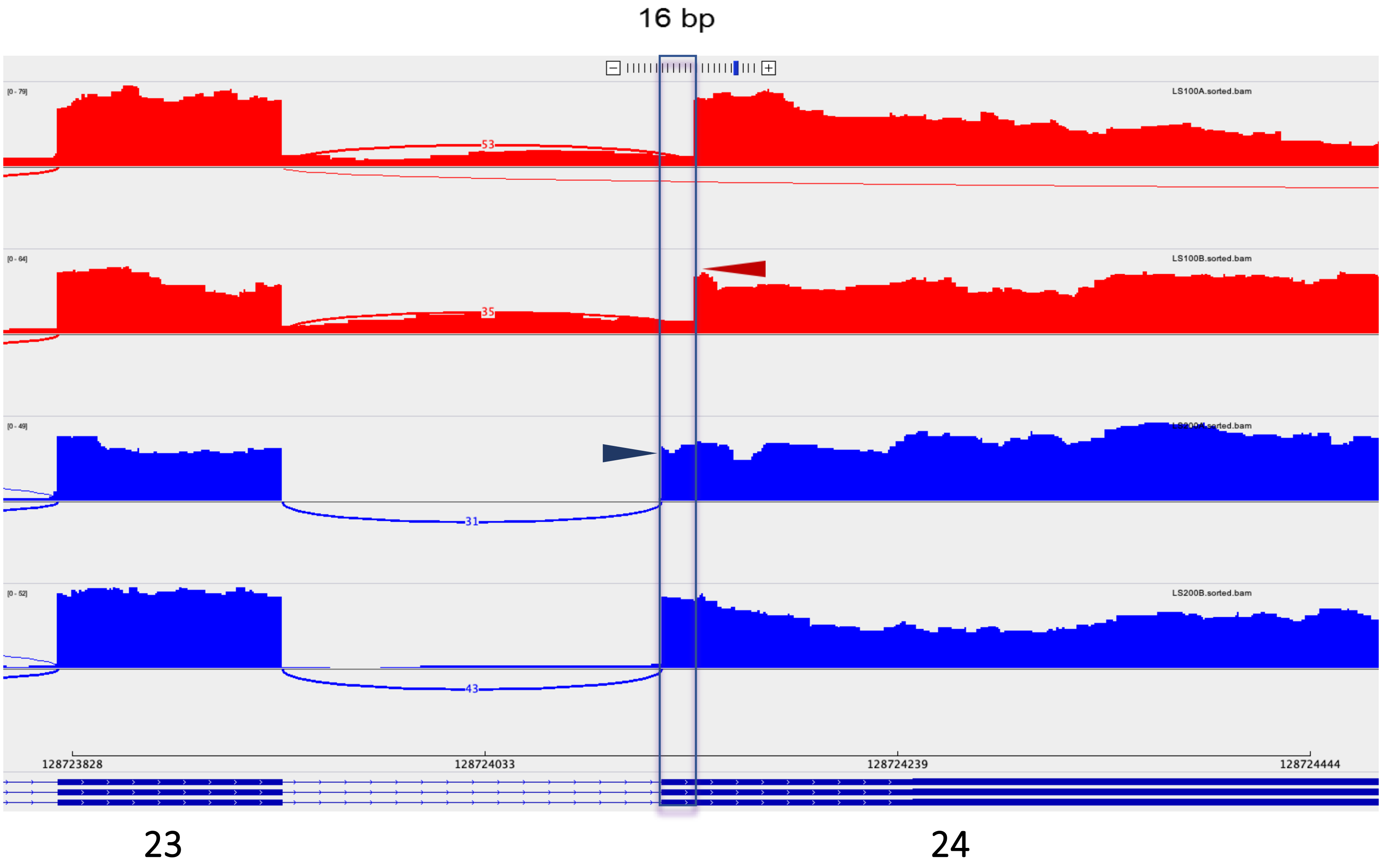

As a result of a mutation in the canonical splice acceptor site in LS100 at the intron23/exon 24 junction, the normal splice acceptor is bypassed and a cryptic splice site 16bp into exon 24 is recognized, leading to a frameshift mutation in the distal portion of the mRNA (see Barnes et al, ref 28 for details). The normal splice site in the sibling control is at position 128,724,123 (blue arrow), while the cryptic splice site is at position 128,724,139 (red arrow).

Additional file 4: Figure 3B
Sashimi plot of *OCRL* gene showing exons 20-24
LS300 (red and blue); LS400 (green and brown)

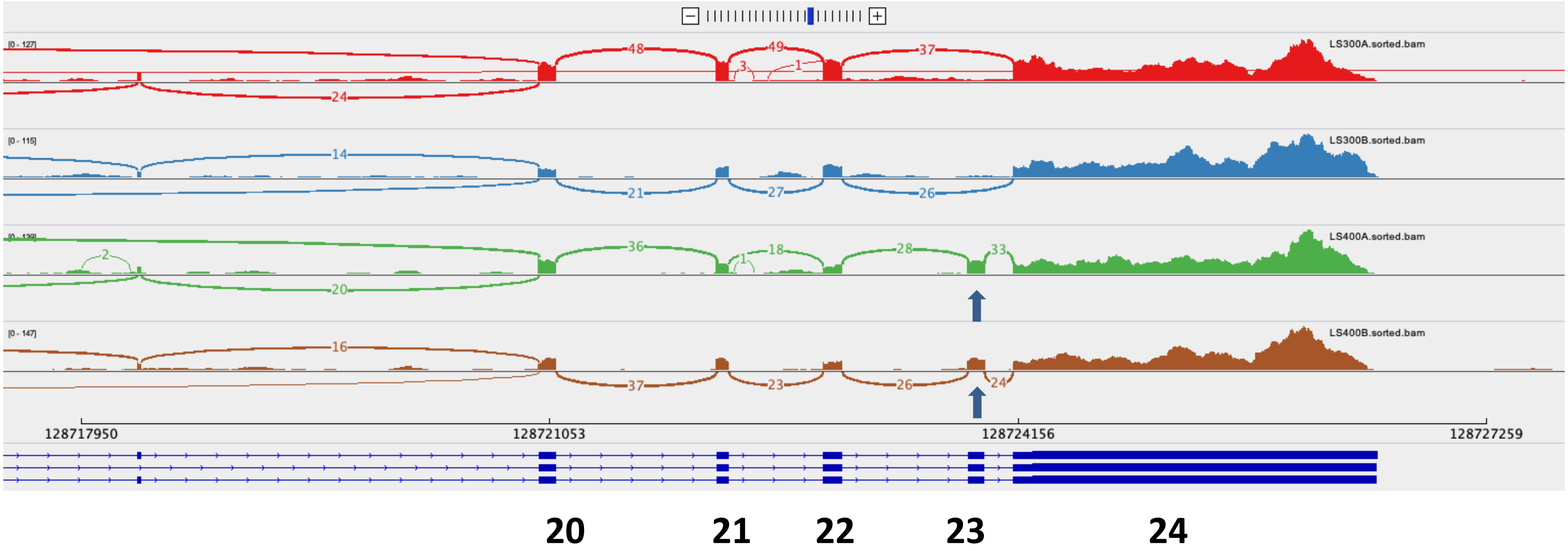

Arrows point to exon 23 in the control, LS400 (bottom two strips). Exon 23 is absent in LS300, the patient sample, as a result of an intron 22 splice acceptor mutation (see Barnes et al., ref 28 for precise cDNA sequence).

Additional file 4: Figure 3C
LS500: note the deletion of a “C” base in exon 20 (black arrow) compared to the LS600 control

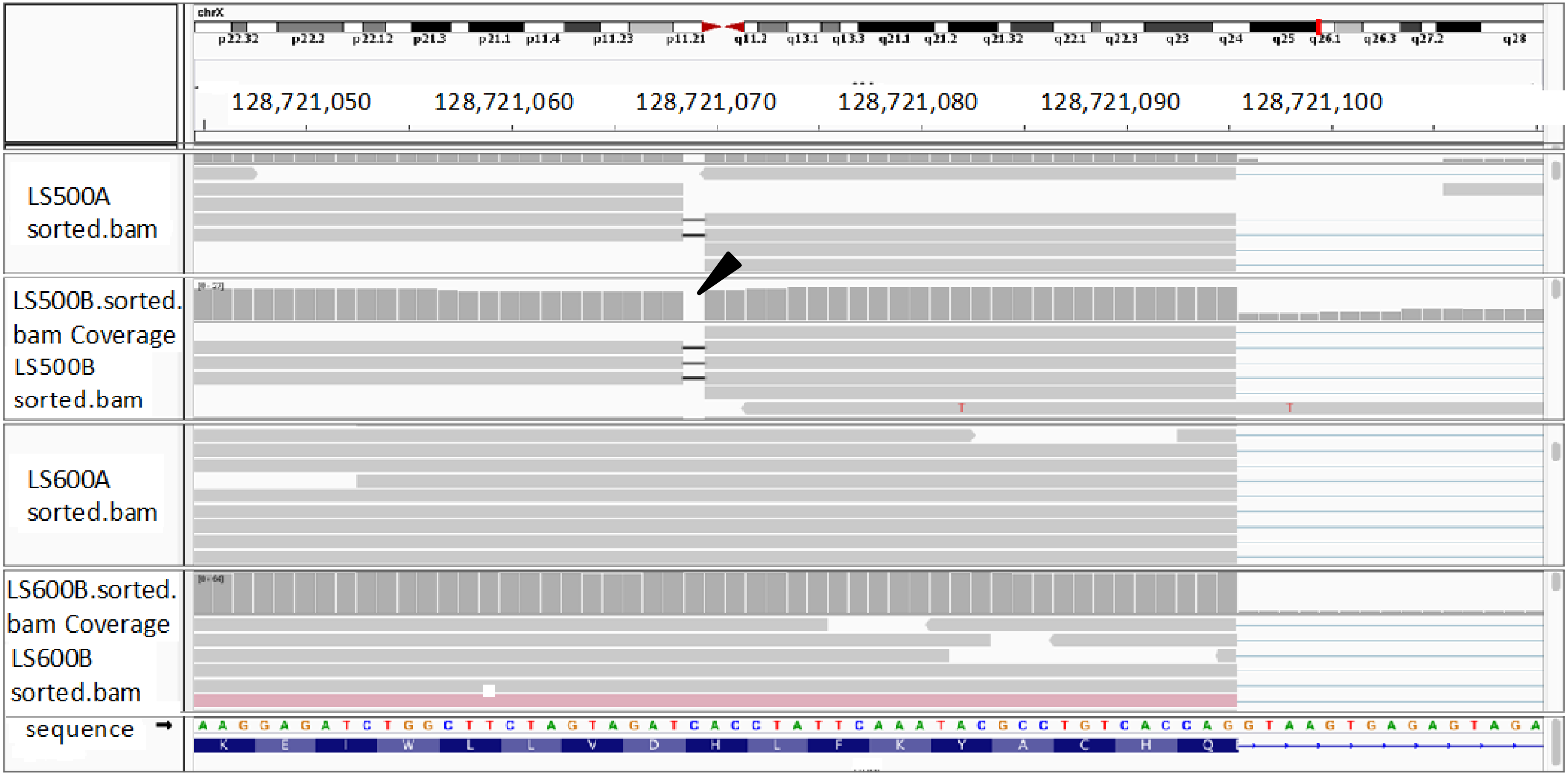

Supplement: Supplementary file 4 — Additional file 4: Figure S3. A and B. Sashimi plots of RNA-seq reads confirming cryptic splice in exon 24 in LS100, and loss of exon 23 in LS200, as described in Barnes et al. C. shows deletion of "C" in exon 20 in LS500. [file 11689_2020_9317_MOESM4_ESM.pdf]
